# Correlation between early dynamics in circulating tumour DNA and outcome from FOLFIRI treatment in metastatic colorectal cancer

**DOI:** 10.1038/s41598-019-47708-1

**Published:** 2019-08-08

**Authors:** Iben Lyskjær, Camilla Skovhus Kronborg, Mads Heilskov Rasmussen, Boe Sandahl Sørensen, Christina Demuth, Mona Rosenkilde, Amanda Frydendahl Boll Johansen, Michael Knudsen, Søren Vang, Søren Rasmus Palmelund Krag, Karen-Lise Garm Spindler, Claus Lindbjerg Andersen

**Affiliations:** 1 Department of Molecular Medicine, Aarhus University Hospital, DK-8200 Aarhus, Denmark; 2 Department of Oncology, Aarhus University Hospital, DK-8200 Aarhus, Denmark; 3 Department of Clinical Biochemistry, Aarhus University Hospital, DK-8200 Aarhus, Denmark; 4 Department of Radiology, Aarhus University Hospital, DK-8200 Aarhus, Denmark; 5 Department of Pathology, Aarhus University Hospital, DK-8200 Aarhus, Denmark; 6 Department of Experimental Clinical Oncology, Aarhus University Hospital, DK-8200 Aarhus, Denmark

**Keywords:** Tumour biomarkers, Chemotherapy

## Abstract

Chemotherapy resistance remains a challenge in the clinical management of metastatic colorectal cancer (mCRC). Here, early changes in cell-free circulating tumour DNA (ctDNA) levels were explored as a marker of therapeutic efficacy. Twenty-four mCRC patients were enrolled and treated with FOLFIRI based first-line therapy. Blood samples collected pre-treatment, at day 7, 14, 21, 60 and at progression were analysed for cell-free DNA (cfDNA) and ctDNA levels using digital droplet PCR. A subset of samples were additionally analysed by targeted sequencing. Patients with high pre-treatment ctDNA or cfDNA levels (≥75^th^ centile) had significantly shorter progression free survival (PFS) than patients with lower levels. Despite an overall decline in ctDNA levels from pre-treatment to first CT-scan, serial analysis identified seven patients with temporary increases in ctDNA consistent with growth of resistant cells. These patients had shorter PFS and shorter overall survival. Targeted sequencing analyses of cfDNA revealed dramatic changes in the clonal composition in response to treatment. Our study suggests that increasing ctDNA levels during the first cycles of first-line FOLFIRI treatment is a predictor of incipient progressive disease and poorer survival. Thus, we demonstrate the importance of monitoring ctDNA levels as early as one week after treatment onset to enable early detection of treatment failure.

## Introduction

Colorectal cancer (CRC) is one of the most frequently diagnosed cancers worldwide, and approximately 20% of all patients present with metastatic disease^1^. The majority of these have incurable spread of disease that requires treatment with palliative chemotherapy^2,3^. Treatment of metastatic CRC (mCRC) is improving, and the median overall survival (OS) for patients presenting with mCRC is currently approaching thirty months in clinical trials^3^. 5-Fluorouracil and leucovorin administered together with Irinotecan (FOLFIRI) has contributed to the improved clinical outcomes of mCRC^4^ and is commonly used as a first- or second-line regimen. Despite treatment improvements, intrinsic and acquired resistance mechanisms ultimately lead to disease progression^5^. At present, the gold standard for assessing treatment effects is the imaging-based Response Evaluation Criteria in Solid Tumours (RECIST)^6^. Although RECIST is instrumental for standardised assessment and communication of radiological management of cancer patients, its current implementation has some limitations. These include lack of reproducibility, difficulties with classification of patients that have multiple or “non-measurable” metastases, and some studies have reported poor correlation with important clinical endpoints, such as progression free survival (PFS) and OS^7^. Hence, new approaches enabling early assessment of chemotherapy efficacy could conceivably lead to early adaptation and hence optimized treatment of mCRC.

Recently, the quantificaion of total cell-free DNA (cfDNA) and circulating tumour DNA (ctDNA) has been proposed as a way to monitor changes in tumour burden^8–12^.

Therefore, the primary aim of this phase II study of mCRC patients treated with FOLFIRI based first-line therapy, was to assess whether changes in plasma cfDNA and ctDNA, during the first months of treatment, could be used as an early marker of FOLFIRI effectiveness.

## Results

### Study pipeline

**Figure 1:**
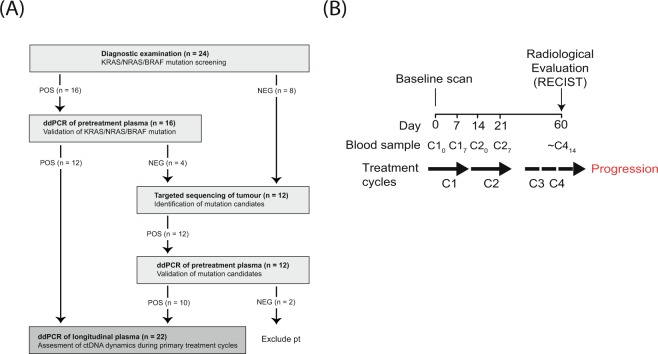
Study design and overview of collected samples. (**A**) Study design and pipeline for selection of patient-specific mutations for ddPCR analysis. Boxes stipulate the method applied and objective at individual analysis steps with POS and NEG indicating a positive or negative result, respectively. The number of patients are shown in parenthesis. (**B**) Overview of collected samples and treatment cycles. ddPCR, digital droplet PCR; CX, treatment cycle X; CXY, treatment cycle X at day Y.

In order to apply a clinically applicable approach we used the following strategy to select patient-specific mutations as ctDNA markers (patient enrolment and study pipeline are presented in Fig. 1A). Tumour samples from all patients were screened for known hotspot mutations in *KRAS*, *BRAF* and *NRAS* as part of routine diagnostic examination. The pre-treatment plasma sample from patients with a tumour hotspot mutation was screened for that mutation by droplet digital polymerase chain reaction (ddPCR) and, if confirmed, the mutation was chosen as a ddPCR marker to monitor plasma ctDNA in longitudinal samples. ctDNA specific ddPCR assays for patients without *KRAS*, *BRAF* and *NRAS* hotspot mutations were developed to match mutation calls from next generation targeted sequencing of biopsies from primary and metastatic lesions.

### Pre-treatment total cfDNA and ctDNA levels in relation to clinicopathological parameters

Twenty-four patients with mCRC were included (Table 1 and Supplementary Table 1). *KRAS*, *NRAS* or *BRAF* mutations had been identified in tumour samples from sixteen of the twenty-four patients (75%), and in twelve of these (75%) the mutation could be verified in the pre-treatment plasma sample by ddPCR. Tumour DNA from the four patients, where the hotspot mutation was not detected, and from the eight patients without known tumour hotspot mutation, were submitted to targeted sequencing. Sequencing revealed a median of eight mutations eligible for ddPCR assay development per tumour. One or two patient-specific ddPCR assays were designed for each patient, and finally, a total of sixteen mutation assays were applied to the pre-treatment plasma samples (Supplementary Table 3). Eleven were detectable, which corresponded to ten of the twelve patients. In total, ddPCR detected mutated DNA in pre-treatment plasma of twenty-two patients (12 with *KRAS*, *BRAF* mutations and 10 patient specific). The two patients without detectable mutated DNA were excluded from all ddPCR analyses.

**Table 1:** Pre-treatment patient characteristics and cfDNA/ctDNA levels in plasma.

Baseline Characteristics	Patients available for total DNA analysis (n = 24)			Patients available for ctDNA analysis (n = 22)		
	No of patients	Alleles per mL plasma (CHR3/gCYC assay) Median (range)	p-value	Patients	Mutated alleles per mL plasma. Median (range)	p-value
Age (median/range)	64.5 (29–81)			64.5 (45–81)		
<Median	11	5437 (876–72286)	0.73	11	69 (8,2–15400)	0.12
>Median	11	4733 (833–43029)		11	513 (44–24231)	
**Gender**						
Female	11	5400 (833–72286)	0.62	10	94 (23–15400)	0.77
Male	13	5457 (876–42783)		12	179 (8–24231)	
**ECOG Performance status**						
0	14	5428 (876–72286)		14	108 (8–15400)	
1	7	5400 (833–16733)	0.37	5	1120 (81–3086)	0.55
2	1	3217	0.67	1	59	0.67
Unknown	2	21386 (7696–35074)	0.5	2	12127 (23–24231)	0.93
**Sites of metastatic disease**						
Liver metastasis	12	19149 (876–72286)	0.04*	12	684 (23–23231)	0.16
No liver metastasis	12	4508 (833–16733)		10	91 (8–3086)	
Lung metastasis	11	5457 (833–43029)	0.50	10	381 (8–24231)	0.58
No lung metastasis	13	5400 (933–72286)		12	104 (23–15400)	
**Mutation status of tumour**						
WT	7	3617 (833–30600)		5	69 (27–513)	
KRAS mut	12	6268 (1857–72286)	0.05*	12	1803 (8–24231)	0.33
NRAS mut	3^a^	16733 (4067–42783)	0.17	3^a^	107 (101–10199)	0.39
BRAF mut	3^a^	2108 (933–4067)	0.65	3^a^	107 (81–109)	0.79
**Number of metastatic sites**						
1	12	4742 (876–72286)		12	91 (23–15400)	
2	10	5400 (833–40629)	0.77	6	817 (8–24231)	0.21
>3	2	36814 (30600–43029)	0.20	4	1652 (59–11242)	0.45
**Treatment efficacy**, **1**. **RECIST**						
Stabil disease (SD)	19	5457 (833–72286)	0.52	17	248 (8–24231)	0.16
Partial/complete response (PR/CR)	5	4067 (2610–5620)		5	44 (27–2486)	
Progression disease (PD)	0			0		

Wilcoxon rank sum test was applied to test for correlations between patient characteristics and cfDNA and ctDNA. ^a^Two patients were excluded (see Fig. 1A) and hence data from n = 22 patients is shown. ^b^One patient (pt109) had a colon tumour with a BRAF mutation and a rectum tumour with a NRAS mutation. p-values < 0.05 are indicated in bold and with *.

ctDNA, circulating tumour DNA; cfDNA, cell-free DNA, ECOG, Eastern Cooperative Oncology Group; RECIST, Response Evaluation Criteria In Solid Tumours.

At last follow up (January 2018) progressive disease had occurred in twenty-one of the twenty-two patients, and twelve patients had died. The median PFS and OS of the cohort (n = 22) was 218 days (95%CI = 197–265) and 315 days (95%CI = 274–373), respectively.

### Pre-treatment cfDNA and ctDNA levels are associated with treatment outcome

**Figure 2:**
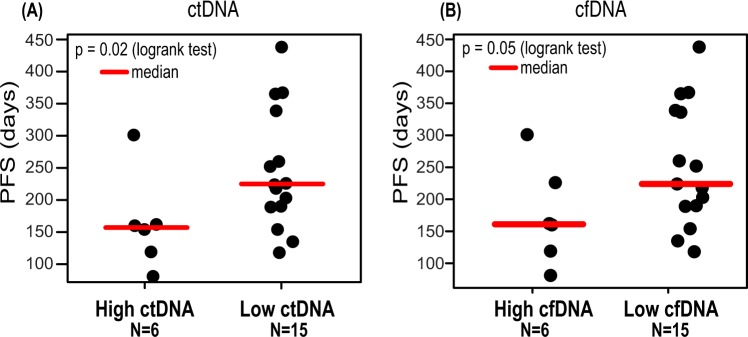
Progression free survival for patients with or without a high pre-treatment level of ctDNA/cfDNA. Patients with a high pre-treatment ctDNA level (**A**) (75^th^ centile) or cfDNA level (**B**) have a shorter PFS than patients with a low level. ctDNA, circulating tumour DNA; cfDNA, cell-free DNA.

The median pre-treatment levels of ctDNA and cfDNA were 108 (range 8–24231) and 5408 (range 833–72286) GEs per mL plasma, respectively. Patients with high ctDNA levels (≥75^th^ centile) at pre-treatment (C1_0_, see Fig. 1B) had a shorter PFS (157 days versus 226 days) than patients with low pre-treatment ctDNA levels (HR = 3.34, 95%CI = 1.20–9.29, p = 0.02) (Fig. 2A). The same was evident for cfDNA (HR = 2.68, 95%CI = 0.96–7.48, p = 0.05) (Fig. 2B).

### ctDNA levels decline from pre-treatment to first response evaluation

Plasma samples were analysed prior to the first treatment cycle (C1_0_), at day seven (C1_7_) during the first cycle, at day zero and seven during the second cycle (C2_0_ and C2_7_, respectively), at first radiological status evaluation (C4_14_) and at the time of clinical validated progression (Fig. 1B). A ctDNA decline from pre-treatment to first response evaluation was observed for all patients (p = 0.001, Wilcoxon signed rank test) (Supplementary Fig. 1.) This was in agreement with RECIST evaluations, which indicated either stable disease (n = 17) or partial or complete response (n = 5) (Table 1). We investigated whether patients with at least a 10-fold reduction in the level of ctDNA from pre-treatment to pre-cycle-two (C2_0_) had a longer PFS, as recently proposed by Tie *et al*.^13^ (Supplementary Fig. 2). We observed a shorter PFS in patients with a ≥ 10-fold reduction in ctDNA (n = 6, HR = 3.20, 95%CI = 1.10–9.20, p = 0.02). As this appeared to conflict with the general assumption that a reduction in ctDNA is reflecting a reduced tumour burden and response to the therapy, we searched for confounders. Thereby, it was revealed that all six patients with ≥10-fold ctDNA reductions had higher pre-treatment ctDNA levels than the median, while this was only the case for 5/15 of the patients with <10-fold ctDNA reductions (Fisher’s Exact Test, p-value = 0.06, odds ratio 8.89).

### Multiple increases in early ctDNA level are associated with decreased PFS

**Figure 3:**
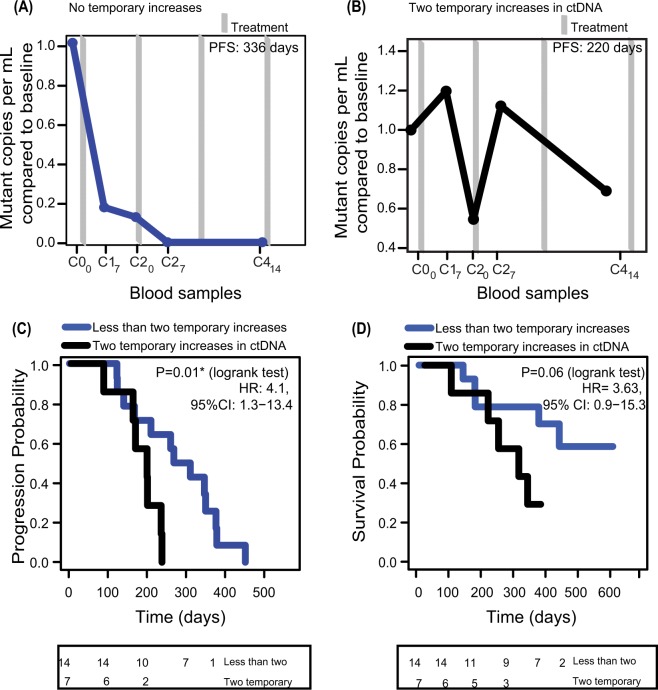
Time to progression for patients with or without two occurrences of temporary increasing ctDNA levels. Patients with multiple occurrences of temporary ctDNA increases have a shorter PFS and OS than patients with stable/decreasing ctDNA levels. For one patient, only C1_0_ and C4_14_ samples were accessible and therefore this patient was excluded from this analysis. (**A**) Representative patient with one temporary ctDNA increase during the first 60 days of treatment. (**B**) Representative patient with two instances of temporary ctDNA increases. (**C**) Progression free survival (PFS). Boxed numbers represents the number of patients in each group not having clinical progression at the given timepoint. (**D**) Overall survival (OS). Boxed numbers represents the number of patients in each group alive at the indicated timepoint.

Despite the overall trend of declining ctDNA levels towards the first response evaluation (Supplementary Fig. 1), ctDNA levels nevertheless fluctuated during or between treatment cycles. Longitudinal ctDNA levels for all patients are given in Supplementary Fig. 3. In seven patients, we observed two occurrences of temporary ctDNA level increases, *i*.*e*. a higher ctDNA level compared to the previous drawn blood sample, during the first 60 days of treatment. We speculated if these temporary raises could indicate growth of resistant cells and potentially would be associated with a shorter time to progression. In agreement with this hypothesis the mean PFS of the seven patients was significantly shorter than for the rest of the patients (p = 0.01, HR = 4.11, 95%CI = 1.27–13.36) (Fig. 3C). Their OS were correspondingly shorter (p = 0.06, HR = 3.63, 95%CI = 0.87–15.25) (Fig. 3D).

### Targeted sequencing of pre-treatment cfDNA samples identifies mutations present at time of treatment initiation

In order to determine if alterations selected on basis of results from the diagnostic examination were as informative as those selected from tumour sequencing, we investigated if the chosen markers were increasing at time of clinical progression (n = 10 samples available). Only four of seven diagnostic work-up ctDNA markers increased at progression, while all three mutations identified by sequencing increased (Table 2). We speculated whether sequencing of the cfDNA directly would be an even better approach for identifying mutations suited for monitoring of treatment response. To address this, the pre-treatment plasma remnants after the former analyses were sequenced using a custom gene panel (Nugen Target Enrichment) targeting the genomic regions most frequently mutated in CRC (Supplementary Table 5). Pre-treatment plasma was available for 17 of the 24 patients, two of which no ctDNA marker had previously been identified. Often only small plasma volumes were left and therefore the average target coverage was limited for some samples – median 246x (range 100–419x). Matching germline samples were also subjected to sequencing using the same gene panel. Genomic variants were identified in all of the pre-treatment samples. On average 59 variants were identified per sample (range 2–153, Supplementary Table 6), including the two patients where no ctDNA marker previously had been identified (pt106 and pt108). Generally, a good concordance was observed between the ddPCR results and the targeted sequencing results for the 14 ddPCR markers covered by the plasma sequencing panel (Table 3). Four ddPCR-detected markers were not detected by plasma-sequencing. For three of these, ddPCR indicated minor allele frequencies (MAF) below 2%. At the position of these mutations the sequencing coverage was only 57x, 5x and 125x (mean for whole panel was 112x, 195x, and 310x), potentially explaining why these variants were not detected. One mutation (ctDNA marker for pt117) was not identified by sequencing (coverage 109x) even though ddPCR analysis indicated a MAF of 37%. Importantly, other mutations were identified by plasma sequencing in these four patients. Furthermore, often the mutation selected for ddPCR was not the most abundantly observed by plasma sequencing. In conclusion, sequencing of plasma could in all cases have been used for analyses of changes in ctDNA levels in response to therapy.

**Table 2:** Evaluation of whether mutations applied as ctDNA markers for ddPCR are informative or non-informative at progression.

Patient	ctDNA fold change^a^	Informative^b^	Days to progression^c^	Mutation detected by ddPCR at progression	Source of selection of ctDNA marker for ddPCR
pt101	46	Yes	6 (252)	Yes	Diagnostic examination
pt103	0.04	No	35 (154)	Yes	Diagnostic examination
pt104	12	Yes	15 (301)	Yes	Sequencing of primary tumor
pt105	38	Yes	10 (135)	Yes	Diagnostic examination
pt109	164	Yes	5 (365)	Yes	Sequencing of liver metastasis
pt113	8	Yes	0 (260)	Yes	Sequencing of lung metastasis
pt115	16	Yes	19 (189)	Yes	Diagnostic examination
pt116	30	Yes	38 (119)	Yes	Diagnostic examination
pt118	0.8	No	1 (160)	Yes	Diagnostic examination
pt119	0.9	No	9 (162)	Yes	Diagnostic examination

Informative versus non-informative mutations applied as ctDNA markers for ddPCR. ctDNA fold change is measured as ctDNA change from first status evaluation to sample nearest progression. An informative mutation is defined as a mutation increasing towards progression.

^a^ctDNA change from first status evaluation to sample nearest progression.

^b^Sample was considered informative if an increase in ctDNA levels were observed towards progression.

^c^Samples collected up to 38 days before progression were considered progression samples.

**Table 3:** Concordance between ddPCR and plasma-sequencing analysis of pre-treatment plasma samples.

Pt^a^	Mutation for ddPCR analysis	Mutation present in sequencing of pre-treatment sample	MAF% detected by sequencing	Mean coverage of cfDNA sequencing	MAF% detected by ddPCR
pt101	KRAS G12D	no	Not available	112	1.7
pt102	TP53–221T < TTC	yes	0.3	406	3.8
pt103	KRAS G12D	yes	28	306	36
pt104	APC-507C > T	yes	40	275	49
pt105	BRAF V600E	yes	9	419	2.7
pt107	KRAS G12D	yes	30	238	39
pt109	PARK2-214G > A	Not on panel	Not on panel	211	17
pt110	KRAS G12C	yes	17	152	79
pt111	KRAS G12V	no	Not available	195	0.7
pt112	ERBB2-392A > G	yes	4	327	7.4
pt113	TP53-003G > A	no	Not available	310	0.8
pt114	Tp53-533G > A	yes	13	253	1.7
pt116	KRAS G12A	yes	33	310	36
pt117	KRAS G12V	no	Not available	109	37
pt118	KRAS G13D	yes	36	170	38

Targeted sequencing of pre-treatment plasma sample. For 17 out of 24 patients plasma DNA were available for targeted sequencing. NA means that no sample within two months of progression was available.

^a^No mutated DNA in patients pt106 and pt108 were detected.

### Therapy induced selection pressure dramatically changes the clonal composition of ctDNA

**Figure 4:**
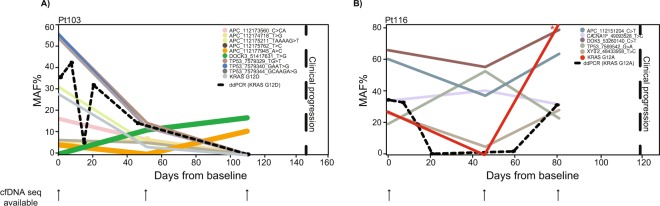
Targeted sequencing of longitudinal cfDNA from two patients. (**A**,**B**) Targeted sequencing was performed on cfDNA from patient 103 and 116 (pre-treatment, first status evaluation and a progression sample - collected within 40 days of clinical progression). Several mutations were detected in all samples. Shown are mutations detected in two or more samples. *Due to low coverage (<5x) in the specified sample the reported MAF may be an overestimate.

In this study we used a one marker strategy for monitoring therapy response. As it can be seen from Table 2, the risk of this strategy is that the selected marker may turn out to be subclonal, and while informative of the initial response, it may be non-informative of progression. Therefore, we hypothesized that a strategy based on monitoring multiple mutations would be better because this would increase the chance of including an informative marker. To address this, we compared our one-marker ddPCR and our targeted cfDNA sequencing strategies. For patients 103 and 116 sufficient plasma cfDNA from pre-treatment, first status evaluation and progression samples were available for analysis with both ddPCR and targeted sequencing (Fig. 4). It was evident from sequencing of cfDNA from patient 103 that treatment onset changed the composition of ctDNA dramatically (Fig. 4A). Sequencing of cfDNA from patient 116 revealed that even though the *KRAS* mutation chosen for ddPCR analysis was informative of progression (Table 3), it was not the most optimal mutation to monitor (Fig. 4B). The *TP53* G > A mutation was increasing already after the first time point indicating that the treatment was not effective already at the first status scan evaluation. This knowledge could have been used to change treatment prior than at clinical progression.

In summary, targeted sequencing of cfDNA gives a panel of mutations that can be monitored, thus giving a better indication of which mutations that are clonal and therefore, believed to be informative, in contrast to ddPCR where prior knowledge of mutational status often is a request. Consequently, direct sequencing of cfDNA will probably be the best approach for the future.

## Discussion

Identification of patients that do not benefit from chemotherapy is of great importance, both to reduce side effects of ineffective treatments, and to facilitate changes in the treatment strategy. In this study, we aimed to investigate whether changes in plasma ctDNA during the first three weeks of treatment were predictive of FOLFIRI efficacy. Our results indicate that FOLFIRI efficacy can be predicted prior to the first response evaluation and opens for rapid revision of the treatment strategy.

Patient and disease characteristics were as expected from other studies on this patient group^14^. However, OS was shorter than reported in recent randomized trials involving mCRC, but comparable to survival in retrospective series on patients receiving combination chemotherapy^15^, as well as to population based cohorts with incurable disease were treatment is merely palliative as is the case for the patients in the current study^16^.

We found that high pre-treatment ctDNA levels were negatively correlated to outcome. This is in line with previous observations^13,17^. Equally high total cfDNA at diagnosis correlated to shorter PFS, as previously reported^18,19^. We did not find any correlation between the number or type of metastasis and the ctDNA levels (data not shown).

Recently, it has been suggested that a large reduction in the ctDNA level shortly after initiation of treatment is a potential marker of treatment effect^13,17^. Tie *et al*. investigated the ctDNA levels in a cohort consisting of 47 patients treated with 5-fuorouracil and oxaliplatin and four treated with FOLFIRI and found a trend towards longer PFS for patients with a 10-fold, or higher, ctDNA reduction (p = 0.27, 95%CI = 0.6–5.6)^13^. We could not confirm this trend in our study, neither with a ten-fold reduction threshold nor other thresholds tested. It should be noticed that both studies include small cohorts, and therefore the results should be taken with caution. Moreover, Tie *et al*. investigate samples collected at different time points than in the current study.

In our study, we analysed 103 serial plasma samples collected between pre-treatment and first RECIST evaluation for twenty-two patients (overview of all analysed plasma samples are given in Suppl. Table 7). Uniquely, we measured ctDNA levels as early as one, two and three weeks after treatment start. The ctDNA levels were surprisingly dynamic in this window, with several temporary increases in ctDNA (Supplementary Fig. 3). Seven patients even showed two temporary increases in ctDNA. Speculating if the increasing ctDNA levels could reflect growth of resistant cells, we compared PFS in these patients relative to the remaining patients and found that they had a significantly shorter PFS, and shortened OS. In agreement with these findings, Garlan *et al*., recently reported that mCRC patients with increasing ctDNA levels between the pre-treatment sample and the second or third treatment cycle had significant shorter PFS and OS^20^. Thus, by early serial ctDNA analysis it appears to be possible to predict which patients have minimal benefit of the given therapy, potentially providing an early opportunity to revise the treatment strategy.

In the current work, a single mutation was used for ctDNA monitoring of each patient. We chose this strategy in order to make it clinically applicable, robust and affordable. A weakness of the one-marker strategy is that it is essential to select an ancestral mutation representative of all tumour cells. If subclonal, the results may potentially lead to incorrect conclusions. Our analyses, from ten patients with samples available close to progression, revealed that sequencing of the tumour samples facilitated selection of more informative mutations as compared to the diagnostic examination. In the clinic, *RAS/RAF* mutational status is determined in order to direct the choice of treatment, and do not involve quantification. Consequently, we could not determine the likelihood of a mutation being subclonal or clonal on basis of the results from the diagnostic examination. From the tumour sequencing on the other hand, we selected mutations on basis of the minor allele frequency and driver potential, and thus increased the chance of selecting a clonal mutation for ddPCR analysis.

We hypothesized that applying a multi-target strategy would be better. Through targeted plasma sequencing we identified mutations in all patients – and further confirmed the ctDNA levels determined by ddPCR. We next hypothesised that targeted sequencing of longitudinally samples would give a better indication of progression than mutations selected on basis of tumour sequencing of diagnostic examination. To investigate this, we applied targeted sequencing directly to longitudinal cfDNA samples from a small number of patients. These analyses indicated the major effects of chemotherapeutics on clonal composition. From sequencing of samples from patient 103 it was evident that some mutations, including the one chosen for ddPCR analysis, were not informative of progression, as these clones apparently were eliminated by the applied therapy. However, an *APC* (12177945_A > C) mutation appeared subclonal at baseline and increased towards progression. Additionally, a *DOCK3* mutation that was not detected in the pre-treatment sample emerged during the first cycles of therapy, and seemed to be part of one of the clones driving resistance to therapy and thus progression. Thus, mutations expected to be clonal, based upon sequencing of the pre-treatment sample, proved to be non-informative of progression as other clones evolved during the applied selection pressure. This stresses the need to not only monitor more than one mutation, but also to do it unbiased, *e*.*g*. without prior knowledge, as new mutations do arise during treatment.

The targeted sequencing analysis of patient 103 indicated the possibility of there being more than one tumour lesion present in this patient as the mutations emerging towards progression (*APC* A > C and *DOCK3*) did not have any ancestral clones in common with the rest of the identified mutations, which seemed to respond to the applied therapy.

Our study has some limitations. Firstly, the modest cohort size inherently makes the confidence intervals of our findings wide. Secondly, the use of only one single patient-specific ctDNA marker may limit the sensitivity towards detecting ctDNA and impose the risk that the selected marker is non-ancestral and hence may turn out non-informative of future resistant clone(s). Thirdly, the chosen method for cfDNA sequencing analysis yielded relatively low sequencing coverage levels, thus making it difficult to detect low frequency alterations. In future studies, multiplexing strategies, *e*.*g*. based on targeted massive parallel sequencing may limit such a risk as both indicated by the findings presented in this article and by others^21–23^. Theoretically, multiplexing will also increase the sensitivity for detecting ctDNA^24,25^.

In conclusion, very early evaluation of ctDNA changes during therapy may be beneficial for patients through rapid detection of treatment failure. Additional studies are needed to thoroughly assess the potential clinical benefit.

## Materials and Methods

### Patients and collection of blood samples

This prospective, observational phase II study recruited patients undergoing treatment for mCRC at the Department of Oncology, Aarhus University Hospital, Denmark. Inclusion criteria were age ≥18, non-resectable, histopathological verified mCRC, with indication for first-line FOLFIRI-based treatment, and measurable disease according to RECIST version 1.1^6^. Patients were treated according to Danish National Guidelines. Chemotherapy included: Leucovorin (400 mg/m^2^ intravenous infusion) and Fluoruracil (400 mg/m^2^ bolus infusion or 2400 mg/m^2^ continuous infusion/46 hours), Irinotecan 180 mg/m^2^ and some RAS/RAF wildtype (WT) patients got additionally Panitumumab 6 mg/kg or Cetuximab 500 mg/kg, every two weeks (overview of treatment and patient characteristics is given in Supplementary Table 1). Treatment decisions were made after each four treatment cycles based on CT-scanning of the chest and abdomen/pelvic areas and evaluation according to RECIST version 1.1. The study was approved by the The Central Denmark Region Commitees on Health Research Ethics (M-2014-298-14) and the Danish Data Protection Agency. Written informed consents were obtained from the patients.

PFS and OS were defined as time from first day of treatment until the date of clinical progression (RECIST evaluation) or death from any cause, respectively. Patients who did not experience an event were censored by the last date of observation.

### Isolation and quantification of DNA

Within two hours of the blood draw, EDTA Plasma (median 3.5 mL, range 1.5–4.0) was isolated from 10 mL of blood by centrifugation at 1400 g for 20 min at 20 °C. DNA was extracted from formalin-fixed, paraffin-embedded (FFPE) tumour tissue using QiAamp DNA FFPE tissue kit (Qiagen). Tumour DNA was quantified using Qubit (Thermofischer) and stored at −80 °C. Leucocyte DNA was extracted with the QIAamp DNA Blood Midi Kit (control DNA). Cell free plasma DNA (cfDNA) was extracted using the QIAamp Circulating Nucleic Acid Kit (Qiagen). The isolated cfDNA was eluted in 60 μL elution buffer and stored at −80 °C. Quantification of cfDNA was done using ddPCR as previously described^8^, and is reported as copies per mL plasma or genomic equivalents (GE). We used a B-cell immunoglobulin rearrangement specific ddPCR assay^26^ to identify cfDNA samples with contaminating lymphocyte DNA. These samples were excluded as contamination is an indication of undesired cell lysis.

### DNA quantification by ddPCR

ctDNA was detected and quantified using ddPCR. The experiments were conducted according to the ddPCR MIQE-guidelines (Supplementary Table 2)^27^. Serial plasma DNA samples were analysed on a QX200 AutoDG Droplet Digital PCR System according to the manufacturer’s instructions (Bio-Rad). Each analysis included a positive, a negative (matched leucocyte DNA) and a non-template control (H_2_O). The positive control sample was either a matched tumour DNA or a ~150 bp template DNA oligo of the mutated sequence. The ddPCR reaction volume was 22 µL, consisting of 13 µL mastermix (18 mM forward and reverse primer, 0.05 mM probe, 2x Supermix for probes (no UTP) (Cat. No. 186–3023; Bio-Rad)) and 9 µL cfDNA. Data were analysed using QuantaSoft v1.6.6 software (Bio-Rad). Thresholds for separating positive and negative droplets were determined based on the positive and negative control samples on each plate. Mutated copies per mL plasma were calculated and used as a measure of concentration. Both previously reported and novel mutation specific ddPCR assays were used (Supplementary Table 3). The novel assays were designed using Primer3^28^ and included two probes to quantify both the mutated and the WT alleles. Amplicon length was, when possible, kept below 100 nucleotides. Limit of detection (LOD) and limit of blank (LOB) for each assay (Supplementary Table 3) were determined using a panel of 24 negative control samples from healthy donors as described previously^29^. For all plasma samples reported to be negative for the assessed patient-specific mutation, a minimum cfDNA input of 700 GEs were analysed (median 1303 GEs).

### Targeted sequencing of tumour DNA

DNA from tumours without known hotspot mutations in *KRAS*, *BRAF*, or *NRAS* were subjected to targeted sequencing using a previously reported hybridization capture panel (Roche Nimblegen) covering 410 cancer genes^30,31^. An overview of the sequenced tumour samples is presented in Supplementary Table 4. Tumour and matched normal DNAs were fragmented (Covaris E220) and subjected to library preparation using the KAPA Hyper Library Preparation Kit (KAPA Biosystems). The indexed libraries (twelve tumour and normal DNA pairs) were pooled and paired-end sequenced using Illumina® NextSeq Medium v2, 300 bp flow cells. Adapter sequences were trimmed using Trim Galore (Babraham Bioinformatics), and mapped to the hg19 reference genome using BWA MEM (v. 0.7.5)^32^. Picard MarkDuplicates was used to inspect the alignment and remove duplicates. Mapping in areas with INDELs were refined using GATK IndelRealigner^33^ and systematic errors in base quality scores were identified and adjusted using GATK BaseRecalibrator^33^. Somatic SNVs and INDELs were called using a combination of GATK MuTect2^34^ and VarScan2^35^.

Patient specific somatic point mutations to be used for ddPCR assay development were selected based on following criteria: Minimum 30 reference reads in the tumour sample (median tumour coverage was 526x), should be among the most clonal mutations in the tumour as indicated by a high allele frequency, and the alternative allele frequency should be below 0.002 in the germline sample. Additionally, mutations in known CRC driver genes were preferred (such as *APC*, *TP53*).

### Targeted sequencing of circulating-free DNA

We performed targeted sequencing using a commercial version of anchored multiplex PCR (AMP) (NuGEN). Libraries were prepared from 3–129 ng (median 49 ng) DNA according to manufacturer’s instructions. A custom Nugen Target Enrichment panel was designed (NuGEN). The panel covers 28622 bases including: (1) the 127 most frequently mutated regions in CRC patients identified from TCGA data^36^, (2) five most frequently mutated regions identified by Giannakis *et al*.^37^ and not identified by TCGA, (3) all of the coding regions of *TP53* and *APC*, and 4) 18 ID-SNPs (Supplementary Table 5). Unique identifiers (UIDs) are included as part of the adapter design for error correction. Libraries were subjected to 21 cyclers of PCR amplification. Pools were single-end sequenced using Illumina® NextSeq Medium v2, 150 bp flow cells, with 15 base index reads to ensure sequencing of the region containing the UIDs. Data was handled and processed as previously described^38^. A random 6 nucleotide unique molecular identifier (UMI) were identified in each read, before the reads were mapped to the human hg19 reference genome using BWA MEM (v. 0.7.5)^32^. Barcodes that had mutated during PCR amplification, or contained sequencing errors, were rescued using UMI-tools group (v. 0.4.0)^39^ with the directional method and edit distance set to 1. Rescue of erroneous UMIs increases both sensitivity and specificity, as well as hinders inflation of the estimated number of unique molecules sequenced. Consensus families were obtained using a custom script by collapsing reads with the same rescued UMI barcode and genomic position. Consensus families with less than three reads were discarded. For each position a consensus base was obtained by requiring 55% similarity across all reads in a family, otherwise an N was inserted. Consensus sequencing quality scores were generated using the mean of individual quality scores per base in each family. Consensus reads were mapped to hg19 using BWA MEM and variants identified using MuTect2 (GATK v. 3.7) using default settings. All reported mutations were accepted as confident by Mutect2 (PASS variants). The median unique coverage were 253x (range: 109–419x).

### Statistical analysis

Wilcoxon rank sum test was applied to test association between patient characteristics and cfDNA and ctDNA. Survival data were analysed by the Kaplan–Meier method, and differences between the groups were estimated by the log-rank test. Analyses were performed using R statistical software^40^ and R packages survival and rms^41,42^. A p-value ≤ 0.05 was considered statistically significant. Effect sizes are indicated by 95% confidence intervals.

## Supplementary information

Supplementary Figures

Supplementary Tables 1-7

## Data Availability

The data from findings of this study are available from the corresponding author upon reasonable request.
