# Supplementary material for: Correlation between early dynamics in circulating tumour DNA and outcome from FOLFIRI treatment in metastatic colorectal cancer
Source: Sci Rep. 2019 Aug 8;9:11542. doi: 10.1038/s41598-019-47708-1 (PMC6687711; doi:10.1038/s41598-019-47708-1)

Supplementary information

Correlation between early dynamics in circulating tumour DNA and outcome from FOLFIRI treatment in metastatic colorectal cancer

Iben Lyskjær, Camilla Skovhus Kronborg, Mads Heilskov Rasmussen, Boe Sandahl Sørensen, Christina Demuth, Mona Rosenkilde, Amanda Frydendahl Boll Johansen, Michael Knudsen, Søren Vang, Søren Rasmus Palmelund Krag, Karen-Lise Garm Spindler and Claus Lindbjerg Andersen

Supplementary figure legends

Supplementary Figure 1: Mutant allele frequencies at baseline ($C1_0$), at cycle 1 day 7 ($C1_7$), at cycle 2 day 0 ($C2_0$) and day 7 ($C2_7$), and at first status evaluation

CR: complete response, PR: partial response, SD: stabile disease, PD: progressing disease.

Supplementary Figure 2: Time to progression for patients with or without a ten-fold ctDNA reduction

Kaplan-Meier plot showing the difference in time to progression for patients with or without a ten-fold reduction in the level of ctDNA from pre-treatment to pre-cycle-two ($C2_0$).

Supplementary Figure 3: Longitudinal ctDNA levels from baseline to first status evaluation for the 21 patients

A) The changes in mutant copies per mL of plasma for the seven patients with two temporary increases ctDNA levels, *i.e.* a higher ctDNA level compared to the previous sample (indicated by red lines) is displayed. Day one and two in each treatment cycle, where FOLFIRI are administrated to the patients, are shown as grey vertical lines. (B) The 14 patients with none or only a single ctDNA increase.

Supplementary Figure 4: Representative example of comparison of singleplex and duplex assay

Thermal gradient experiments were performed for all assays on FFPE tumour DNA (or template DNA when tumour DNA was not available) and germline DNA with the aim of determining the optimal amplification conditions during thermal cycling. Furthermore, single and duplex reactions were tested and compared to ensure that duplexing did not influence the performance of the assays.

Supplementary Figure 5: Example of examination of Limit of Blank (LOB) on negative control samples

All assays were applied to 24 negative control samples. Four representative negative samples and one positive control sample are shown here for the TP53-208 mutation and wt assay (run as duplex assay). More information about the assays are supplied in Supplementary Table 3.

Supplementary Figure 6: Example of examination of Limit of Blank (LOB) on negative control samples

All assays were applied to 24 negative control samples. Four representative negative samples and one positive control sample are shown here for the TP53-263 mutation and wt assay (run as duplex assay). More information about the assays are supplied in Supplementary Table 3.

Supplementary Figure 1

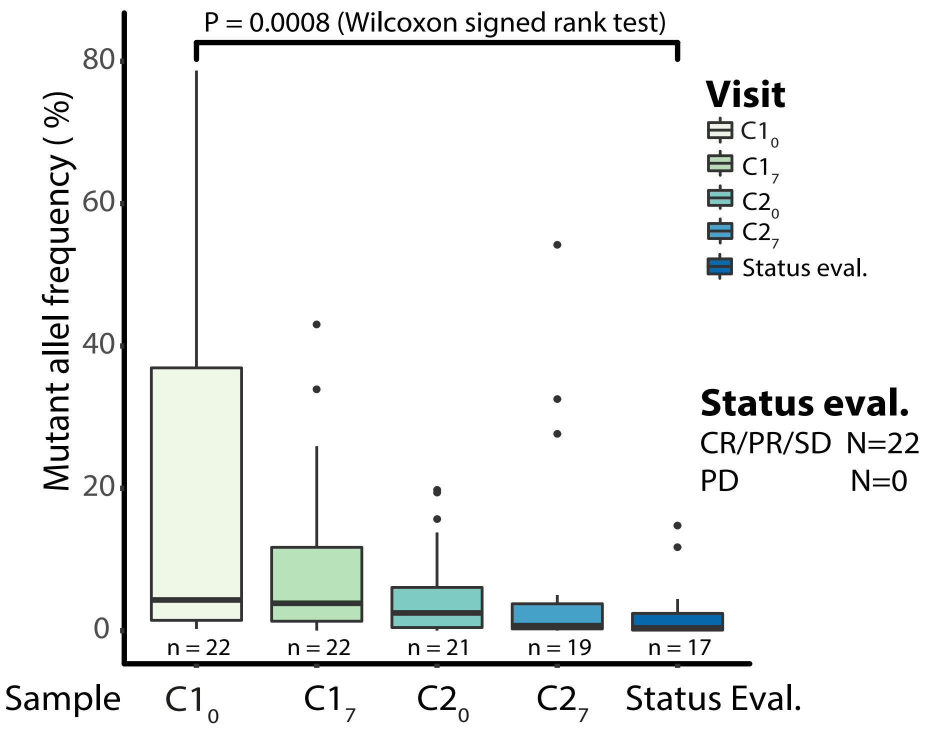

Supplementary Figure 2

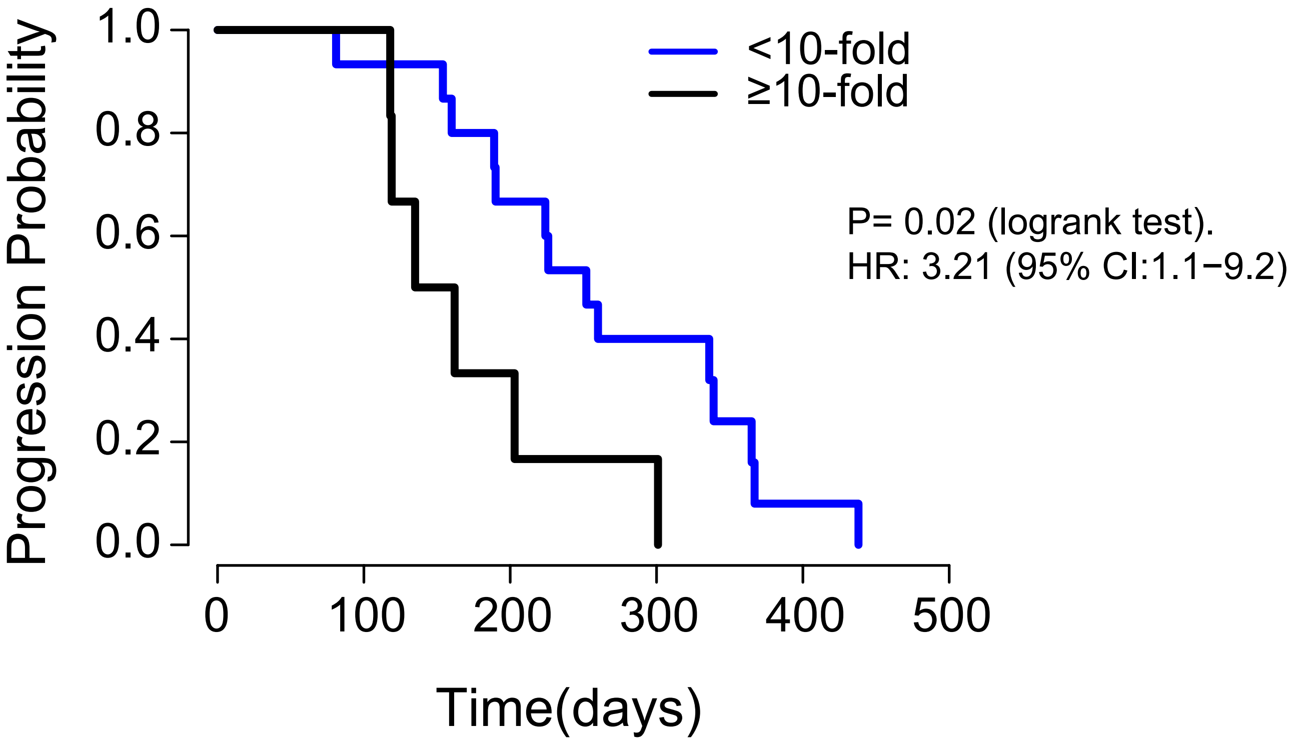

15	14	10	6	1	<10-fold
6	6	2	1		≥10-fold

Supplementary Figure 3

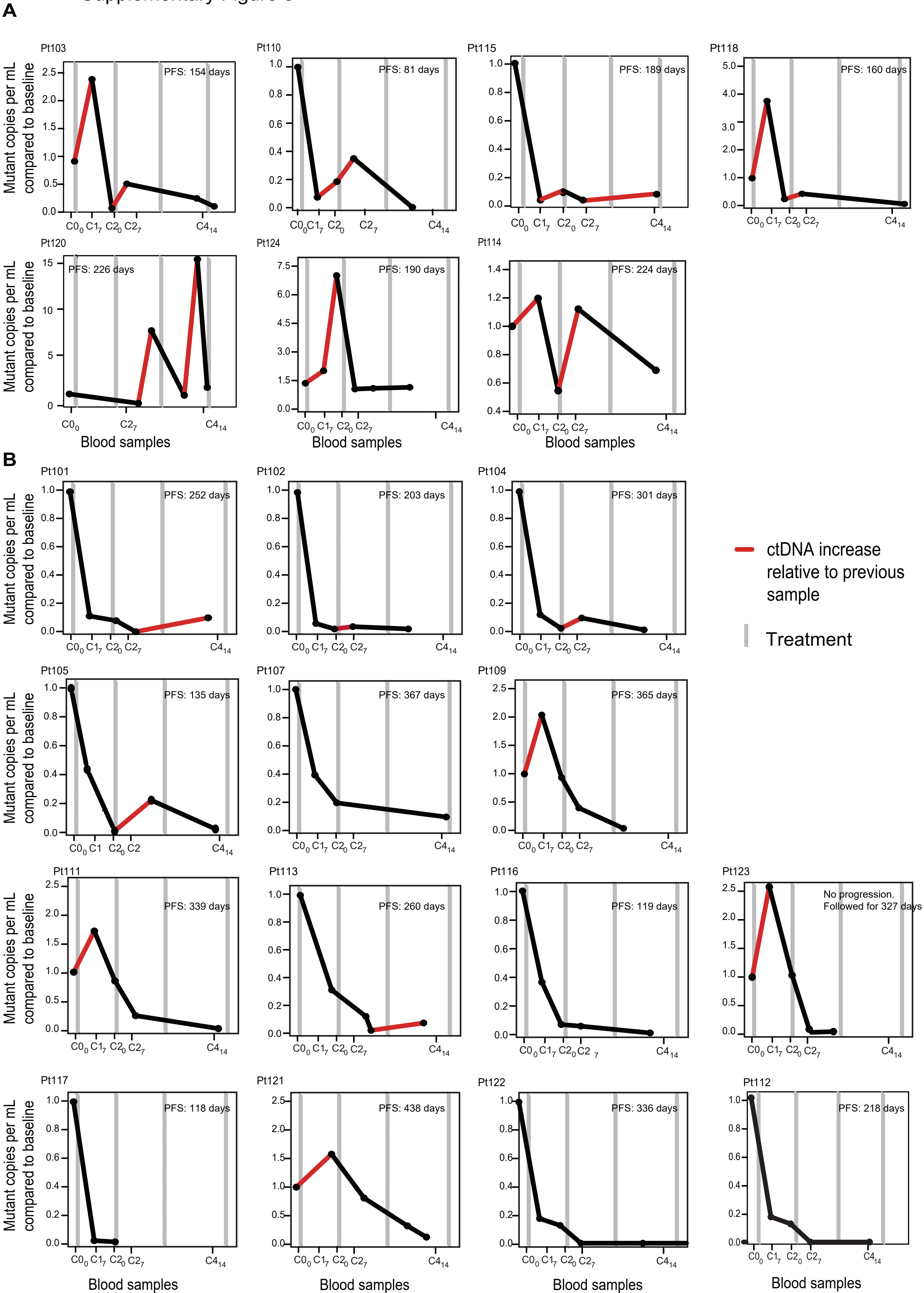

Supplementary Figure 4

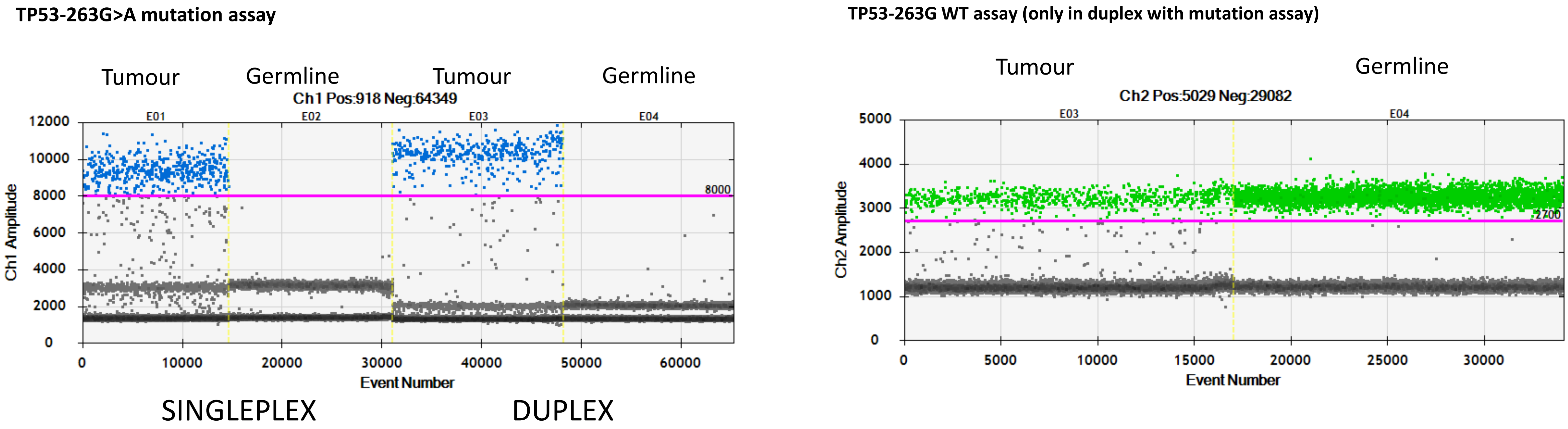

Supplementary Figure 5

TP53-208 mutation assay.

Data from one positive control (template for mutation) and 4 negative controls.

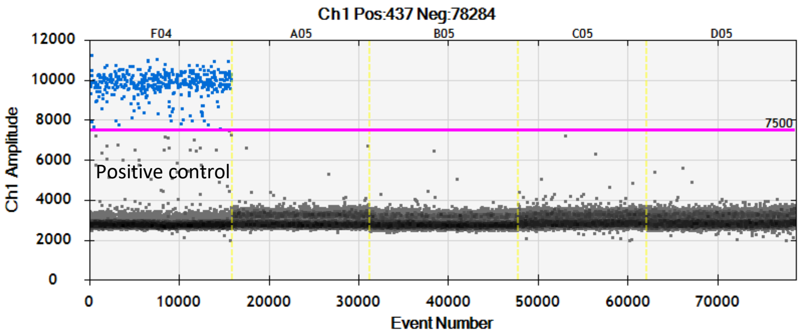

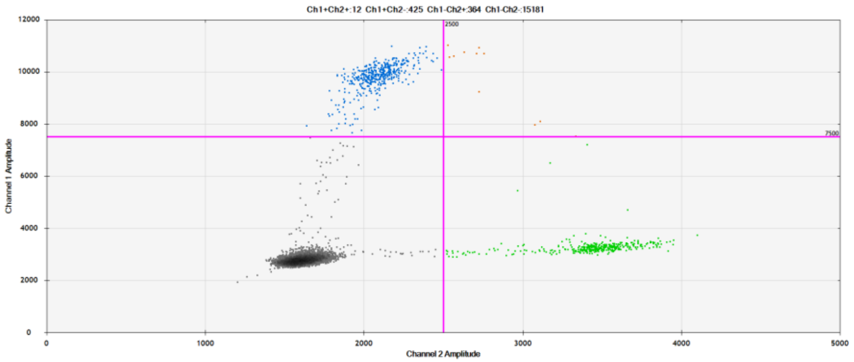

TP53-208 WT assay.

Data from one positive control (template for mutation) and 4 negative controls.

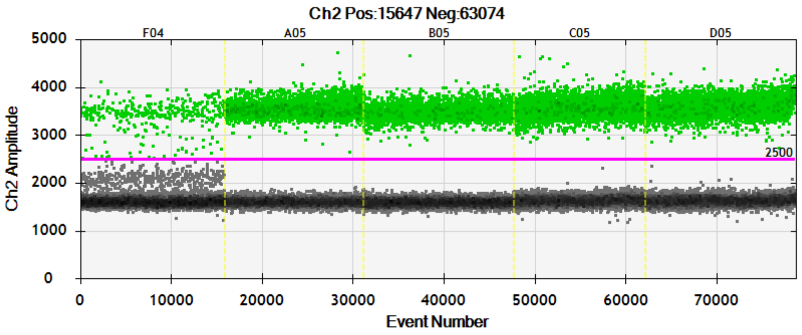

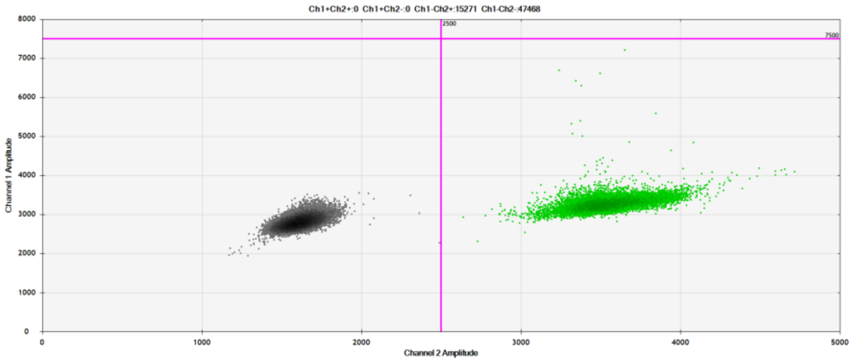

Supplementary Figure 6

TP53-263 mutation assay.

Data from four negative control and one positive controls (tumour DNA).

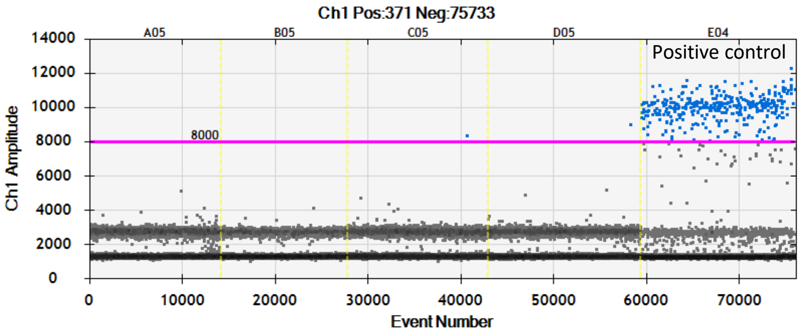

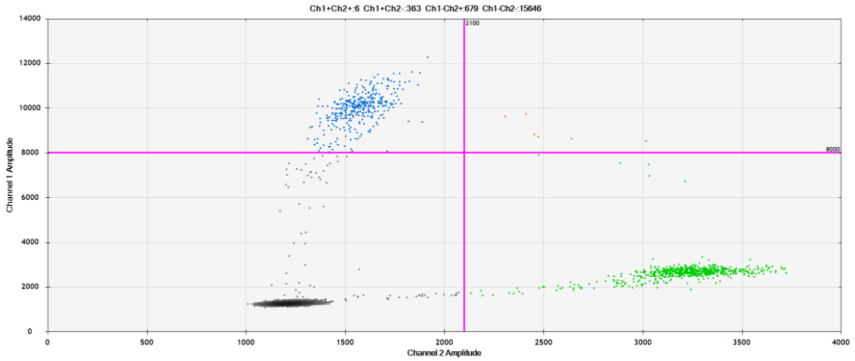

TP53-263 WT assay.

Data from four negative controls and one postive controls (tumour DNA).

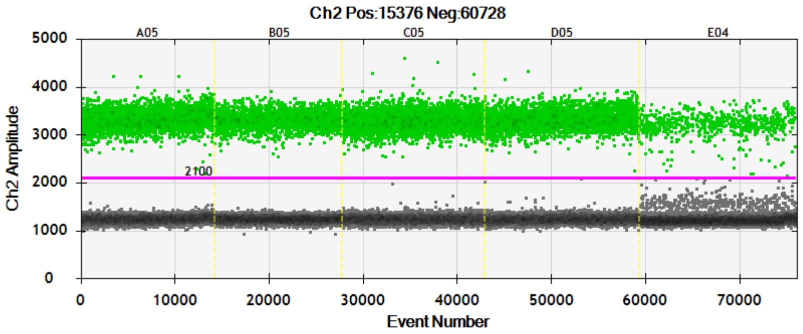

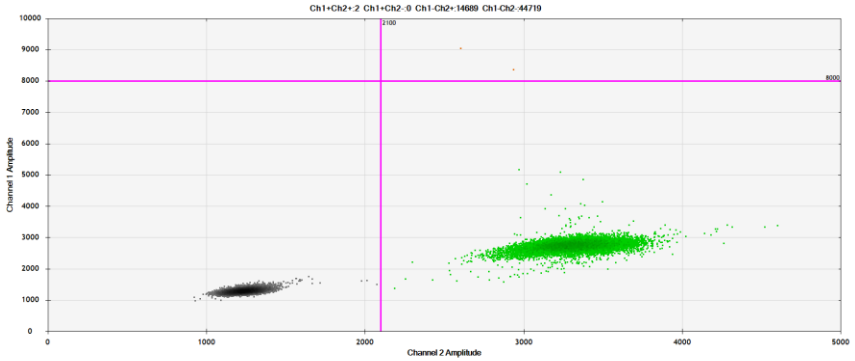

Supplement: Supplementary file 1 — Supplementary Figures [file 41598_2019_47708_MOESM1_ESM.pdf]
